# Alterations of mental defeat and cognitive flexibility during cognitive behavioral therapy in patients with major depressive disorder: a single-arm pilot study

**DOI:** 10.1186/s13104-019-4758-2

**Published:** 2019-11-06

**Authors:** Tomokazu Murata, Yoichi Hiramatsu, Fuminori Yamada, Yoichi Seki, Shinobu Nagata, Takayuki Shibuya, Mizue Yokoo, Remi Noguchi, Mari Tanaka, Keiko Oshiro, Daisuke Matsuzawa, Yoshiyuki Hirano, Eiji Shimizu

**Affiliations:** 1 Research Center for Child Mental Development, Chiba University, 1-8-1 Inohana, Chuo-ku, Chiba-shi, Chiba 260-8670 Japan; 2 Cognitive Behavioral Therapy Center, Chiba University Hospital, 1-8-1 Inohana, Chuo-ku, Chiba-shi, Chiba 260-8670 Japan; 3 Department of Cognitive Behavioral Physiology, Graduate School of Medicine, Chiba University, 1-8-1 Inohana, Chuo-ku, Chiba-shi, Chiba 260-8670 Japan

**Keywords:** Major depressive disorder, Mental defeat, Cognitive flexibility, Cognitive behavioral therapy, Depression, Quality of life, Treatment effects, Intervention effects

## Abstract

**Objective:**

Mental defeat affects the occurrence and chronicity of depression and cognitive flexibility. This study aimed to examine changes in mental defeat and cognitive flexibility scores after cognitive behavioral therapy including IR. In the intervention group, patients with depression (n = 18, mean age = 37.89 years) received 15 cognitive behavioral therapy sessions. Patients completed the Beck Depression Inventory-II; Mental Defeat Scale; Cognitive Flexibility Scale; EuroQol five dimensions questionnaire; Patient Health Questionnaire-9 and seven-item Generalized Anxiety Disorder Scale before the intervention, after six sessions, and post-intervention. The healthy control group (n = 33, mean age = 37.91) completed all scales once and did not receive treatment.

**Results:**

Post-cognitive behavioral therapy, a significant decrease was observed in Beck Depression Inventory-II, Mental Defeat Scale, Cognitive Flexibility Scale, and Patient Health Questionnaire-9 scores. Although mental defeat and cognitive flexibility did not reach the level of the healthy control group, they demonstrated improvement. Therefore, when treating depression, mental defeat and cognitive flexibility should be measured in addition to depressive symptoms.

*Trial registration* This study was registered retrospectively in the national UMIN Clinical Trials Registry on July 25, 2016 (registration ID: UMIN000023320)

## Introduction

Mental defeat describes a state of mentally giving up during the trauma, associated with a perceived loss of dignity, autonomy, sense of being human, free will, and self-esteem [1]. Further, it has been identified as one of the most important peritraumatic predictors of PTSD development and chronification [2]. In addition, mental defeat was associated with the severity and chronicity of depression [3, 4].

Many patients with PTSD experience flashbacks as still and static images as a part of fragmented and disorganized trauma memories and have difficulty distinguishing between images and memories [5]. In the previous study, images are defined as still and static pictures for a very short time, such as flashbacks [5]. Conversely, memories are defined as moving and dynamic pictures for a longer time, such as autobiographical memories, and consist of serial images [5]. Further, intrusive memories are those memories that are “spontaneous involuntary memories of a (mostly) negative event that repeatedly intrude upon consciousness, often against one’s will; they are hard to control and may disrupt one’s ongoing activities” (p. 101) [6]. Patients who experienced psychological trauma repeatedly experience impressions of trauma event through intrusive memory of trauma [7]. Intrusive memories of trauma are related to, not only PTSD but also depression severity [7–9].

In imagery rescripting (IR), the patient imagines the (start of a) traumatic (or otherwise negative) experience and then imagines an intervention that changes the course of events so that a more satisfying outcome is achieved [10]. IR as a treatment or treatment ingredient for a variety of disorders, including PTSD and other anxiety disorders, depression, eating disorders, sleep problems and personality disorders [10, 11]. Further, research has indicated that IR may diminish the severity of intrusive memories in individuals with depression [12, 13] (The continuation of introduction is Additional file 1).

## Main text

### Methods

#### Participants and design

Participants were recruited through clinical referrals and web-based advertisements between February 2014 and March 2016. Calculations of the required sample were based on a power of .80, a significance level of .05, and an expected effect size of 0.8 on depressive symptoms at posttest. The estimations necessitated a sample size of 15 participants. The maximum dropout rate for CBT has been reported as approximately 30% in Japan [14]. With a 30% expected dropout, we guessed a total sample size of 20 for recruits. A total of 20 participants responded, and recruitment efforts ceased, as 20 was the target patient enrollment number. Of the recruited participants, one was determined to be ineligible, and one participant was removed from analyses because the therapist performed only the Beck Depression Inventory-II (BDI-II). Therefore, 18 participants were included in the final analyses (Additional file 2).

All participants met the study’s inclusion criteria: (a) a primary diagnosis of Major Depressive Disorder (MDD; based on the Diagnostic and Statistical Manual of Mental Disorders, 4th edition, text revision) [15], (b) experience a current episode of major depression based on the Mini International Neurological Interview (MINI); (c) received ≥ 8 weeks of pharmacotherapy treatment, and (d) a score over 20 on the BDI–II.

Healthy participants were recruited using flyers posted on public places. Thirty-three healthy people who were age- and sex-matched with the CBT group and who did not have depression (per the MINI) were used as a healthy control group.

This study was a single-arm trial conducted as a preliminary pilot study. It was registered in the National University Medical Information Network (UMIN) Clinical Trials Registry (no. UMIN000023320).

#### Outcomes

We used the Japanese versions of a range of psychometrically tested questionnaires [16–21]. The BDI-II, which was used to measure depression severity, comprises 21 items measured on a 4-point Likert scale (scores range 0–63). Higher scores indicated more severe depression [22]. The Mental Defeat Scale (MDS) used to measure a sense of a failed struggle and loss of social rank and comprises 24 items measured on a 5-point Likert scale (score range 0–96). Higher scores indicated greater mental defeat [23]. The items on the MDS focus on feelings and perceptions of defeat and loss of social standing and include items, such as “I felt defeated,” “I felt that I had sunk to the bottom of the ladder,” and “I felt that I had lost my standing in the world.” Gilbert and Allan state that the defeat scale has no overlapping items with the BDI-II [24], as the BDI-II includes items such as “Sadness,’’ “Guilty feelings,” and “Loss of interest.” No correlation was found from BDI-II and MDS scores using data of pre-CBT. (Additional file 3). The Cognitive Flexibility Scale (CFS) used to measure cognitive flexibility comprises 12 items measured on a 6-point Likert scale (scores range 12–72). Higher scores indicated stronger cognitive flexibility [25]. The EuroQol five dimensions questionnaire (EQ-5D) was used to measure quality of life. It comprises five items measured on a 3-point Likert scale (scores range − 0.111 to 1.000). Higher scores indicated higher QOL [26]. The Patient Health Questionnaire-9 (PHQ-9) used to measure depression severity comprises nine items measured on a 4-point Likert scale (scores range 0–12). Higher scores indicated stronger severe depression [27]. The 7-item Generalized Anxiety Disorder Scale (GAD-7) used to measure anxiety severity comprises seven items measured on a 4-point Likert scale (scores range 0–21). Higher scores indicated more severe anxiety [28].

For those in the CBT group, each questionnaire was administered pre-CBT (before the first session), mid-CBT (after the sixth session), and post-CBT (after the final session). The healthy control group completed the BDI-II, MDS, and CFS once and did not receive any specific intervention.

#### Intervention

Each participant completed 15 weekly, 50-min CBT sessions following the manual. Fujisawa and colleagues developed a culturally adapted, 16-week individual CBT manual for Japanese patients with MDD, based on the traditional model by Beck and colleagues [29, 30]. In this study, we developed a CBT manual that added mental imagery and memory work (using IR) to 16 sessions of traditional CBT for Japanese patients with MDD. The main treatment steps are described in Table 1.

**Table 1:** Therapy overview

Session	Purpose
Pre	
	Assessment
1	Psychoeducation
2	Case conceptualization
3	Goal setting
4	Activity scheduling
5–6	Cognitive restructuring
Mid	
7–8	Imagery rescripting of intrusive images
9	Assertiveness training
10	Problem solving
11–12	Memory rescripting work
13	Schema work
14	Relapse prevention
15	Termination
Post	

Participants completed the CBT intervention at the psychiatric outpatient section at Chiba University Hospital and the therapy room at Chiba University Graduate School of Medicine. This study was the single-arm pilot study. However, because it was an intervention study, we reported our study according to CONSORT guidelines [31] (Additional file 4).

### Statistical analysis

Changes in scale scores at pre-, mid-, and post-CBT were the dependent variables; time was the independent variable. We compared BDI-II, MDS, CFS, EQ-5D, PHQ-9, and GAD-7 values at pre-, mid-, and post-CBT among the intervention group. We also used a repeated-measures analysis of variance (ANOVA) with a Bonferroni adjustment post hoc comparison. The adjusted α value was α = .05/3/6 = .003. Effect sizes were calculated for changes in scale scores between times (Cohen’s d) [32]. We performed a *t*-test to compare the healthy control group’s BDI-II, MDS, and CFS scores to the scores of the intervention group. The adjusted α value was α = .05/3 = .02.

All analyses were conducted using SPSS for Windows version 23 (IBM, Armonk, New York, USA). EQ-5D was not completed on three occasions, and the last-observation-carried-forward method was used for analyses involving this measure. Statistical significance was set at p < .05.

### Results

#### Baseline data

Table 2 presents participants’ demographic and clinical data.

No serious adverse events were recorded during the intervention.

**Table 2:** Participants’ demographic and clinical characteristics at baseline

Variable	Patients	Control group	Statistics
*n* (men/women)	18 (7/11)	33 (13/20)	X^2^(1) = .001, p = .972
Age (years), mean (*SD*)	37.89 (11.74)	37.91 (11.05)	t(49) = .006, p = .995
Marital status, *n* (%)			
Single	10 (55.56)		
Married	8 (44.44)		
Divorced	0 (0)		
Educational background, *n* (%)			
Finished secondary	1 (5.56)		
Finished tertiary	17 (94.44)		
Years of education, mean (*SD*)	15.89 (1.56)		
Employment status, *n* (%)			
Employed full-time	4 (22.22)		
Sick leave from work	2 (11.11)		
Full-time student	0 (0)		
Sick leave from school	1 (5.56)		
Part-time/homemaker	8 (44.44)		
Unemployed	3 (16.67)		
Medical history, *n* (%)	5 (28)		
Comorbid dysthymic disorder, *n* (%) (MINI)	0		
Comorbid manic episode, *n* (%) (MINI)	0		
Comorbid axis I diagnosis, *n* (%) (MINI)			
None (MDD only)	11 (61.11)		
Anxiety disorder	6 (33.33)		
Other (bulimia nervosa)	1 (5.56)		
Age of onset (years), mean (*SD*)	30.33 (8.13)		
Duration of current depressive episode (years)	3.72		
MDD, single episode	9		
MDD, recurrent episode	9		
Current medication, *n* (%)	18 (100)		
Score of pre-CBT			
BDI-II mean (*SD*)	29.89 (5.76)	3.58 (3.38)	
MDS mean (*SD*)	55.61 (19.36)	5.79 (9.41)	
CFS mean (*SD*)	31.89 (8.92)	50.82 (6.93)	

*MDD* major depressive disorder, *SD* standard deviation, *MINI* Mini International Neurological Interview

#### Treatment outcomes

Repeated-measure ANOVAs revealed a significant effect of time on BDI-II. Post-hoc tests showed a significant difference between pre- and post-CBT (Table 3).

**Table 3:** Comparison of pre-, mid-, and post-CBT outcome measure scores

Scale	Pre	Mid	Post	*F*	Pairwise comparisons (*p*)			Condition (Cohen’s *d*)		
	M (*SD*)	M (*SD*)	M (*SD*)		Pre-post	Pre-mid	Mid-post	Pre-post	Pre-mid	Mid-post
BDI-II	29.89 (5.76)	26.56 (10.97)	18.89 (10.5)	*F*(2,34) = 11.98**	.001	0.454	.011	1.30	.38	.72
MDS	55.61 (19.36)	54.5 (20.43)	38.67 (22.27)	*F*(2,34) = 15.75**	.002	1.000	< .001	.81	.06	.74
CFS	31.89 (8.92)	32.78 (7)	39.22 (9.41)	*F*(2,34) = 15.54**	< .001	1.000	.002	.80	.11	.78
PHQ-9	15.72 (3.59)	10.72 (5.52)	8.61 (5.03)	*F*(2,34) = 21.68**	< .001	.001	.122	1.08	1.07	.29
GAD-7	9.67 (7.56)	7.56 (6.33)	6.33 (4.64)	*F*(1.51,25.63) = 4.56*	.064	.295	.356	.74	.48	.27
EQ-5D	0.66 (0.1)	0.66 (0.74)	0.74 (0.12)	*F*(1.45,24.63) = 5.47*	.110	1.000	.024	.73	0.00	.76

Repeated-measures analyses of variance with Bonferroni post hoc tests

*BDI-II* Beck Depression Inventory-II, *MDS* Mental Defeat Scale, *CFS* Cognitive Flexibility Scale, *PHQ-9* Patient Health Questionnaire-9, *GAD-7* 7-item Generalized Anxiety Disorder Scale, *EQ-5D* EuroQol five dimensions questionnaire, *SD* standard deviation

* p < .05, ** p < .01

Repeated-measures ANOVA revealed a significant effect of time on MDS and CFS. Post-hoc tests showed a significant difference between pre- and post-CBT and mid- and post-CBT on the MDS. Additionally, a significant difference between pre- and post-CBT and mid- and post-CBT was also observed for the CFS (Table 3).

Repeated-measures ANOVAs revealed a significant effect of time on the PHQ-9, GAD-7, and EQ-5D. Post-hoc tests showed a significant difference between pre- and post-CBT, and pre- and mid-CBT, PHQ-9 scores (Table 3).

A series of t-test revealed that the BDI-II and MDS scores of the control group were lower than Post-CBT, and the CFS scores were higher (p < .001; Additional file 5).

### Discussion

The current study was a pilot study of a CBT intervention with an including IR component to determine if the intervention improved the depression severity, mental defeat, and cognitive flexibility in a sample of participants with a diagnosis of major depressive disorder. Our results showed a larger change in BDI-II scores than was previously reported in a study of CBT treatment with patients with depression who closely matched this study’s eligibility that included treatment of pharmacotherapy lasting over 6 weeks and having BDI-II scores over 20 [33, 34] (Additional file 6). These findings offer some preliminary evidence that the addition of IR to CBT might increase its treatment effects on depression; however, this possibility should be further research that includes a matched control group of patients receiving CBT without IR along with the CBT with IR intervention group.

Participants reported significant improvements in mental defeat, suggesting that addressing intrusive memories with CBT and IR might improve mental defeat. To the best of our knowledge, this study is the first in which a CBT intervention was used to address mental defeat in patients with depression; thus, the observed changes in the CBT group cannot be compared with previous results. However, CBT including IR appears to be effective in improving mental defeat in those with depression based on the observed effect size (d = .81) in this study.

Additionally, patients with depression reported increasing cognitive flexibility following the intervention (d = .80). The observed rates of improvement were somewhat larger than a previous CBT intervention with a sample of older adults with depression (mean age = 66.73; d = .51) [35]. Therefore, CBT may be effective for the improvement of cognitive flexibility of adulthood. Further, cognitive flexibility has been shown to be positively correlated with self-reflection [36], and we guess that improving cognitive flexibility will facilitate depression treatment.

Mental defeat scores at post-CBT were higher than those reported in the healthy control group in this study and the previous study [23], and the cognitive flexibility scores were lower [17, 25] (Additional file 6). As the current study does not include follow-up data, it is difficult to reach firm conclusions in this regard. To better understand recurrence prevention and treatment, recovery should be observed to healthy control levels to shed more light on this finding.

While we did observe improvements in BDI-II scores from pre- to post-CBT; however, EQ-5D scores were not significantly different between pre- and post-CBT (from 0.66 to 0.74), consistent with one previous study (from 0.63 to 0.75) [37]. Another study reported a significant improvement in EQ-5D scores over 6 months of pharmacotherapy (from 0.47 to 0.66) [38] (Additional file 6). Perhaps changes in QOL require more time to demonstrate improvements; thus, future studies with follow-up assessments several months after treatment are needed.

The average pharmacotherapy history of depressed patients participating in this study was 3.72 years. Previous research has suggested that those with treatment-resistant depression are more vulnerable to perceiving life stressors as trauma and to have reduced psychological defense [39]. In future research, we should be examined that the effect of IR on depressive symptoms, mental defeat, and cognitive flexibility for not treatment-resistant patients with depression.

### Conclusions

Although the mental defeat and cognitive flexibility of patients with depression did not reach the healthy control level, we showed that the scores of mental defeat and cognitive flexibility change concurrently with the improvement of depression through CBT.

## Limitations

This study has several limitations. First, we used a single-arm trial design, and there was limited data, reducing the generalizability of our results. Future research should attempt to replicate our findings with a larger sample using MDS and CFS. Second, some of the participants had comorbid conditions. Future interventions should employ a stricter control of these confounding variables, or evaluate their influence on patient outcomes. Third, no long-term follow-up data were collected. The collection of such data in future research would permit the examination of long-term effects, reoccurrence risks, and the exploration of the optimal treatment target cut-off scores. Finally, future research should utilize a randomized controlled trial design.

## Supplementary information

**Additional file 1.** Continuation of introduction.

**Additional file 2: Figure S1.** Flowchart of recruited participants and dropouts. CBT, Cognitive behavioral therapy.

**Additional file 3.** Correlations between BDI-II and MDS. *p < .05.

**Additional file 4.** CONSORT 2010 checklist.

**Additional file 5.** BDI-II, MDS, CFS scores Post-CBT and Healthy control group. BDI-II, Beck Depression Inventory-II; MDS, Mental Defeat Scale; CFS, Cognitive Flexibility Scale; SD, standard deviation.

**Additional file 6.** Comparison of the scores of the BDI-II, MDS, CFS and the EQ-5D. BDI-II, Beck Depression Inventory-II; MDS, Mental Defeat Scale; CFS, Cognitive Flexibility Scale; EQ-5D, EuroQol five dimensions questionnaire; SD, standard deviation. *The effect size cannot be calculated because SD is not shown. And, because it is a drug therapy, it is baseline-pharmacotherapy and last visit-pharmacotherapy, not pre-CBT and post-CBT.

## Data Availability

Data generated from or analyzed in the current study are available from the corresponding author upon request.

## Abbreviations

BDI-II: Beck Depression Inventory-II
CBT: cognitive behavioral therapy
CFS: Cognitive Flexibility Scale
EQ-5D: EuroQol Five Dimensions Questionnaire
GAD-7: 7-item Generalized Anxiety Disorder Scale
IR: imagery rescripting
MDS: Mental Defeat Scale
PHQ-9: Patient Health Questionnaire-9
