# Supplementary material for: Alterations of mental defeat and cognitive flexibility during cognitive behavioral therapy in patients with major depressive disorder: a single-arm pilot study
Source: BMC Res Notes. 2019 Nov 6;12:723. doi: 10.1186/s13104-019-4758-2 (PMC6833291; doi:10.1186/s13104-019-4758-2)
Supplement: Supplementary file 1 — Additional file 1. Continuation of introduction. [file 13104_2019_4758_MOESM1_ESM.docx]

Taylor et al., (2011) stated that initial stressors trigger perceptions of defeat and the concomitant activation of an involuntary defeat strategy (IDS), which has cognitive, behavioral, and affective components. Perceiving the inability to avoid or solve these stressors then contributes to the development of depressive symptoms and maintains the sense of defeat [4]. In the present study, we hypotheses that cognitive behavioral therapy (CBT) including IR may improve mental defeat in patients with depression.

Cognitive flexibility refers to “the readiness with which the person’s concept system changes selectively in response to appropriate environmental stimuli” (p.405) [40]. A previous study using CBT to improve cognitive flexibility among older people (mean age = 66.73) with depression [35]. However, no study has examined the effects of CBT, including IR, on the cognitive flexibility of adults with depression (mean age < 60). Therefore, we examined if CBT including IR among adult patients with depression alter mental defeat and cognitive flexibility.

**References**

1. Taylor PJ, Gooding P, Wood AM, Tarrier N. The role of defeat and entrapment in depression, anxiety, and suicide. Psychol Bull. 2011;137:391–420. https://doi.org/10.1037/a0022935.
2. American Psychiatric Association. Diagnostic and statistical manual of mental disorders, 4th ed, text revision. Washington, DC: American Psychiatric Association; 2000.
3. Scott WA. Cognitive complexity and cognitive flexibility. Sociometry. 1962;25:405. https://doi.org/10.2307/2785779.
