# Supplementary material for: Alterations of mental defeat and cognitive flexibility during cognitive behavioral therapy in patients with major depressive disorder: a single-arm pilot study
Source: BMC Res Notes. 2019 Nov 6;12:723. doi: 10.1186/s13104-019-4758-2 (PMC6833291; doi:10.1186/s13104-019-4758-2)
Supplement: Supplementary file 2 — Additional file 2: Figure S1. Flowchart of recruited participants and dropouts. CBT, Cognitive behavioral therapy. [file 13104_2019_4758_MOESM2_ESM.pptx]

## Slide 1
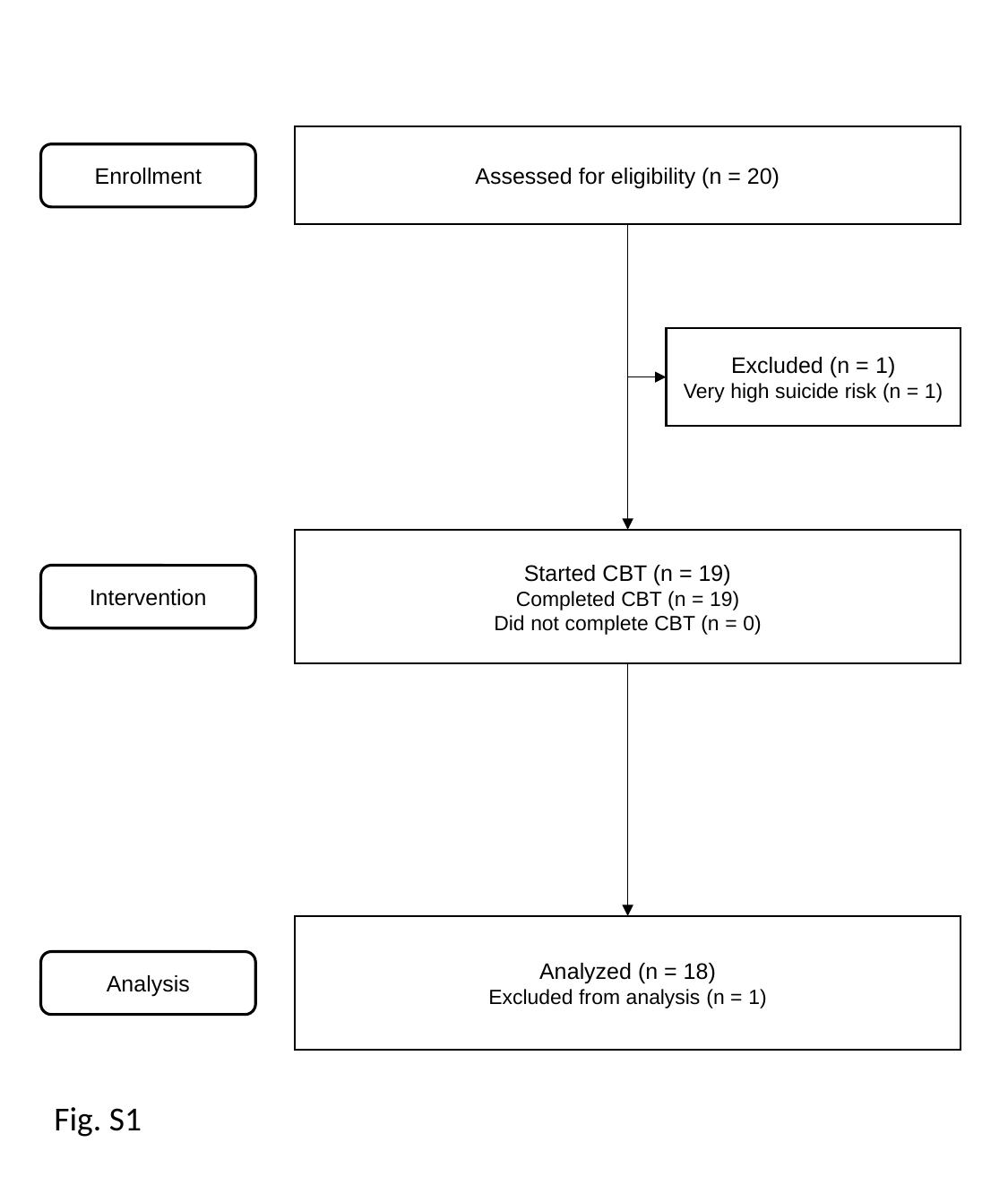

Assessed for eligibility (n = 20)
Enrollment
Excluded (n = 1)
Very high suicide risk (n = 1)
Started CBT (n = 19)
Completed CBT (n = 19)
Did not complete CBT (n = 0)
Intervention
Analyzed (n = 18)
Excluded from analysis (n = 1)
Analysis
Fig. S1
