# Supplementary material for: Alterations of mental defeat and cognitive flexibility during cognitive behavioral therapy in patients with major depressive disorder: a single-arm pilot study
Source: BMC Res Notes. 2019 Nov 6;12:723. doi: 10.1186/s13104-019-4758-2 (PMC6833291; doi:10.1186/s13104-019-4758-2)
Supplement: Supplementary file 3 — Additional file 3. Correlations between BDI-II and MDS. *p < .05. [file 13104_2019_4758_MOESM3_ESM.docx]

| **Additional File 3.** Correlations between BDI-II and MDS | | |
| --- | --- | --- |
|  | BDI-II | MDS |
| BDI-II | - | .389 |
| MDS |  | - |
| *Note.* ^*^*p* < .05  BDI-II, Beck Depression Inventory-II; MDS, Mental Defeat Scale. | | |
