# Supplementary material for: Alterations of mental defeat and cognitive flexibility during cognitive behavioral therapy in patients with major depressive disorder: a single-arm pilot study
Source: BMC Res Notes. 2019 Nov 6;12:723. doi: 10.1186/s13104-019-4758-2 (PMC6833291; doi:10.1186/s13104-019-4758-2)
Supplement: Supplementary file 5 — Additional file 5. BDI-II, MDS, CFS scores Post-CBT and Healthy control group. BDI-II, Beck Depression Inventory-II; MDS, Mental Defeat Scale; CFS, Cognitive Flexibility Scale; SD, standard deviation. [file 13104_2019_4758_MOESM5_ESM.docx]

**Additional File 5** BDI-II, MDS, CFS scores Post-CBT and Healthy control group

|  | Post-CBT | Healthy control |  |  |  |
| --- | --- | --- | --- | --- | --- |
| Scale | M (*SD*) | M (*SD*) | *t* | *p* | Cohen's *d* |
| BDI-II | 18.89 (10.5) | 3.58(3.38) | 6.02 | <.001 | 2.26 |
| MDS | 38.67 (22.27) | 5.79(9.41) | 5.98 | <.001 | 2.17 |
| CFS | 39.22 (9.41) | 50.82(6.93) | 5.02 | <.001 | 1.47 |

Abbreviations: BDI-II, Beck Depression Inventory-II; MDS, Mental Defeat Scale; CFS, Cognitive Flexibility Scale; SD, standard deviation.
