# Supplementary material for: Alterations of mental defeat and cognitive flexibility during cognitive behavioral therapy in patients with major depressive disorder: a single-arm pilot study
Source: BMC Res Notes. 2019 Nov 6;12:723. doi: 10.1186/s13104-019-4758-2 (PMC6833291; doi:10.1186/s13104-019-4758-2)
Supplement: Supplementary file 6 — Additional file 6. Comparison of the scores of the BDI-II, MDS, CFS and the EQ-5D. BDI-II, Beck Depression Inventory-II; MDS, Mental Defeat Scale; CFS, Cognitive Flexibility Scale; EQ-5D, EuroQol five dimensions questionnaire; SD, standard deviation. *The effect size cannot be calculated because SD is not shown. And, because it is a drug therapy, it is baseline-pharmacotherapy and last visit-pharmacotherapy, not pre-CBT and post-CBT. [file 13104_2019_4758_MOESM6_ESM.docx]

| **Additional File 6** Comparison of the scores of the BDI-II, MDS, CFS and the EQ-5D | | | | | | |
| --- | --- | --- | --- | --- | --- | --- |
| Scale | Study | Sample type | Pre-CBT mean (SD) | Post-CBT mean (SD) | ES | Nonclinical |
| BDI-II | Scott et al. (2000) | Depression | 21.7 (7.7) | 13.8 (9.6) | 0.92 |  |
|  | Kennedy et al. (2003) | Depression | 22.7 (8.6) | 19.9 (10.3) | 0.3 |  |
|  | Present study | Depression | 29.9 (5.8) | 18.9 (10.5) | 1.3 |  |
| MDS | Present study | Depression Nonclinical | 55.61 (19.36) | 38.67 (22.27) | 0.81 | 5.79 (9.41) |
|  | Tang et al. (2007) | Nonclinical |  |  |  | 17.9 (20.6) |
| CFS | Johnco et al. (2014) | Depression and anxiety | 50.05 (7.9) | 53.64 (6.08) | 0.51 |  |
|  | Present study | Depression Nonclinical | 31.89 (8.92) | 39.22 (9.41) | 0.8 | 50.82 (6.93) |
|  | Martin et al. (1995) | Nonclinical |  |  |  | 55.5 (6.3) |
|  | Oshiro et al. (2016) | Nonclinical |  |  |  | 46.3 (7.7) |
| EQ-5D | Høifødt et al. (2013) | Depression | 0.63 (0.23) | 0.75 (0.17) | 0.24 |  |
|  | Sobocki et al. (2007)^*^ | Depression | 0.47 | 0.66 | - |  |
|  | Present study | Depression | 0.66 (0.1) | 0.74 (0.12) | 0.73 |  |
| BDI-II, Beck Depression Inventory-II; MDS, Mental Defeat Scale; CFS, Cognitive Flexibility Scale; EQ-5D, EuroQol five dimensions questionnaire; SD, standard deviation. * The effect size cannot be calculated because SD is not shown. And, because it is a drug therapy, it is baseline-pharmacotherapy and last visit-pharmacotherapy, not pre-CBT and post-CBT. | | | | | | |
